# Photonic-chip assisted correlative light and electron microscopy

**DOI:** 10.1038/s42003-020-01473-4

**Published:** 2020-12-07

**Authors:** Jean-Claude Tinguely, Anna Maria Steyer, Cristina Ionica Øie, Øystein Ivar Helle, Firehun Tsige Dullo, Randi Olsen, Peter McCourt, Yannick Schwab, Balpreet Singh Ahluwalia

**Affiliations:** 1 Department of Physics and Technology, UiT The Arctic University of Norway, 9019 Tromsø, Norway; 2 Cell Biology & Biophysics Unit, European Molecular Biology Laboratory, 69117 Heidelberg, Germany; 3 Department of Medical Biology, UiT The Arctic University of Norway, 9019 Tromsø, Norway

**Keywords:** Lab-on-a-chip, Microscopy

## Abstract

Correlative light and electron microscopy (CLEM) unifies the versatility of light microscopy (LM) with the high resolution of electron microscopy (EM), allowing one to zoom into the complex organization of cells. Here, we introduce photonic chip assisted CLEM, enabling multi-modal total internal reflection fluorescence (TIRF) microscopy over large field of view and high precision localization of the target area of interest within EM. The photonic chips are used as a substrate to hold, to illuminate and to provide landmarking of the sample through specially designed grid-like numbering systems. Using this approach, we demonstrate its applicability for tracking the area of interest, imaging the three-dimensional (3D) structural organization of nano-sized morphological features on liver sinusoidal endothelial cells such as fenestrations (trans-cytoplasmic nanopores), and correlating specific endo-lysosomal compartments with its cargo protein upon endocytosis.

## Abstract (web summary)

Tinguely et al. develop a photonic chip-based correlative light-electron microscopy system to generate co-registered multi-modal total internal reflection fluorescence microscopy (TIRF) and electron microscopy (EM) images of biological samples at nanometer scale.

## Introduction

Correlative light and electron microscopy (CLEM) are widely used techniques, from light microscopy on living samples^1^ to on-section CLEM^2^. It has been performed on chemically fixed^3^, as well as frozen^4^ samples, and scales from virus^5^ up to cells in mouse brain^6^. Light microscopy (LM) benefits from the availability of a large variety of contrast mechanisms to collect functional, dynamic and specific information using targeted fluorescence labeling methods. Moreover, LM has a larger FOV as compared to EM enabling screening of large sample sets ensuring capturing of localized events. This includes biological questions regarding rare events (e.g., extravasating metastasizing cancer cells^6^ or plasma membrane reshaping during endocytosis^7^) or addressing heterogeneous subpopulations^8^. On the other hand, EM brings complementary high-resolution down to nanoscale. For the best results in CLEM experiments, data from different modalities should be registered with the highest possible precision. Although challenging, previous reports have used fiducial markers as reference points for location precision from one microscopy platform to the other^9,10^. Another challenging aspect is the loss of fluorescence upon the use of chemical fixation and heavy metals during EM sample preparation.

**Fig. 1:**
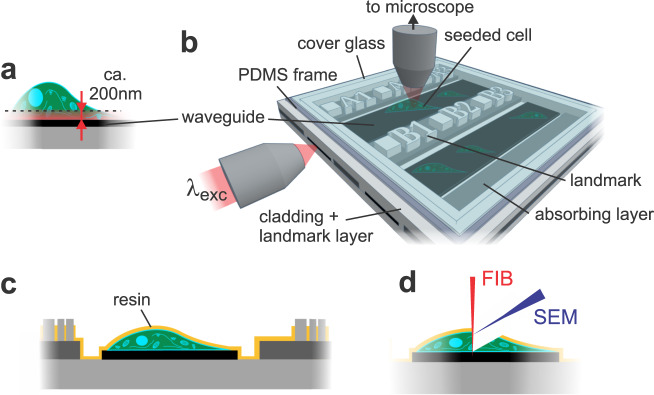
Working principle of chip-based CLEM. **a** The evanescent field of the utilized waveguides reaches approximately 200 nm into the sample from the chip surface. **b** Schematic of chip-based TIRF/dSTORM platform during fluorescence imaging. The biological sample is placed on top of a waveguide and illuminated by the evanescent field created on top of the waveguide surface. Different layers of the waveguide chip including the landmark system are shown. **c** After light microscopy, a thin layer of resin (in yellow) stabilizes the specimen while maintaining landmark visibility for quick position retrieval inside the FIB-SEM. **d** Volume imaging of cell placed on waveguide chip is performed with FIB-SEM.

The photonic-chip, consisting of optical waveguides, was recently introduced as a platform to perform large field of view (FOV) multi-modal optical nanoscopy^11–13^. The sample is hosted directly on top of a waveguide chip and is illuminated by the evanescent field created on top of the waveguide surface, enabling on-chip total internal reflection fluorescence (TIRF) microscopy. Compared to the more conventional epi-fluorescence illumination, the evanescent field excitation significantly improves the axial spatial resolution by only illuminating a thin section of, in the case of waveguides, 100–200 nm (Fig. 1a). Moreover, by removing the fluorescence signal from outside the focal plane, the signal-to-noise ratio is significantly improved, and the phototoxicity as well as photobleaching of fluorophores significantly decreasead^14^. The light into the waveguide is coupled from the side facet of the chip and the fluorescence signal from the sample is captured by any conventional upright microscope. Whereas conventional objective-based TIRF makes use of a single objective for excitation and collection, the chip-based configuration decouples the dependence between the excitation and the collection optics, enabling adjustment-free wavelength multiplexing and free choice of imaging objective without influencing the illumination light path. High intensities (1–10 kW/cm^2^) in the evanescent field can be achieved by fabricating waveguides made of high-refractive index contrast (HIC) materials (e.g., silicon nitride, Si_3_N_4_) and by using thin waveguide geometry (150–220 nm thickness)^11^, enabling a chip-based implementation of direct stochastic optical reconstruction microscopy (dSTORM)^11,15^. The use of HIC materials also enables tight confinement of the light inside the photonic-chip. Moreover, the spatial frequencies of the evanescent field illumination are determined by the refractive index of the material (*n* = 2.05 for Si_3_N_4_) and are thus higher than can be generated even by the oil immersion objective lens (*n* = 1.49). Thus, chip-based microscopy/nanoscopy provides a unique opportunity of imaging large FOV supported by low magnification objective lens while keeping the resolution provided by the high spatial frequencies supported by the HIC waveguide material. These properties were harnessed for chip-based TIRF-structured illumination microscopy (TIRF-SIM)^16^ and light fluctuation based optical nanoscopy, such as entropy based super-resolution imaging (ESI)^11^. Notably, chip-based dSTORM demonstrated an optical resolution of 72 nm over an extraordinarily large FOV, 0.5 × 0.5 mm^2^, and chip-based TIRF-SIM showed a resolution enhancement of 2.4× which surpassed the resolution supported by conventional TIRF-SIM^16^.

Here we extend the utility of the photonic chip illumination towards three-dimensional (3D) CLEM by introducing on-chip landmarks as reference points and by using dyes that are sustained during fixation. Several studies have previously reported CLEM methods combining different super-resolution LM techniques under various conditions, from epifluorescent to TIRF geometries^17–20^. In this work, we combine chip-based TIRF and dSTORM with focused ion beam scanning electron microscopy (FIB-SEM), demonstrating wide-field imaging, super-resolution nanoscopy and 3D EM for cell biology applications using a single photonic chip with a coordinate landmark system.

## Results and discussion

The detailed chip fabrication process can be found in the Supplementary Note 1 “Chip fabrication”. The thickness of the strip waveguides made of Si_3_N_4_ material was 150 nm and the width ranging from 10–1000 µm. Between the waveguides, a 300 nm tall absorbing layer prevents the stray light from neighboring waveguides. A cladding of 1.5 µm SiO_2_ shields the guiding structures from light loss through, e.g., absorbance by crystallized media. At the imaging area, the cladding was removed to lay the cell directly on top of the waveguide surface.

**Fig. 2:**
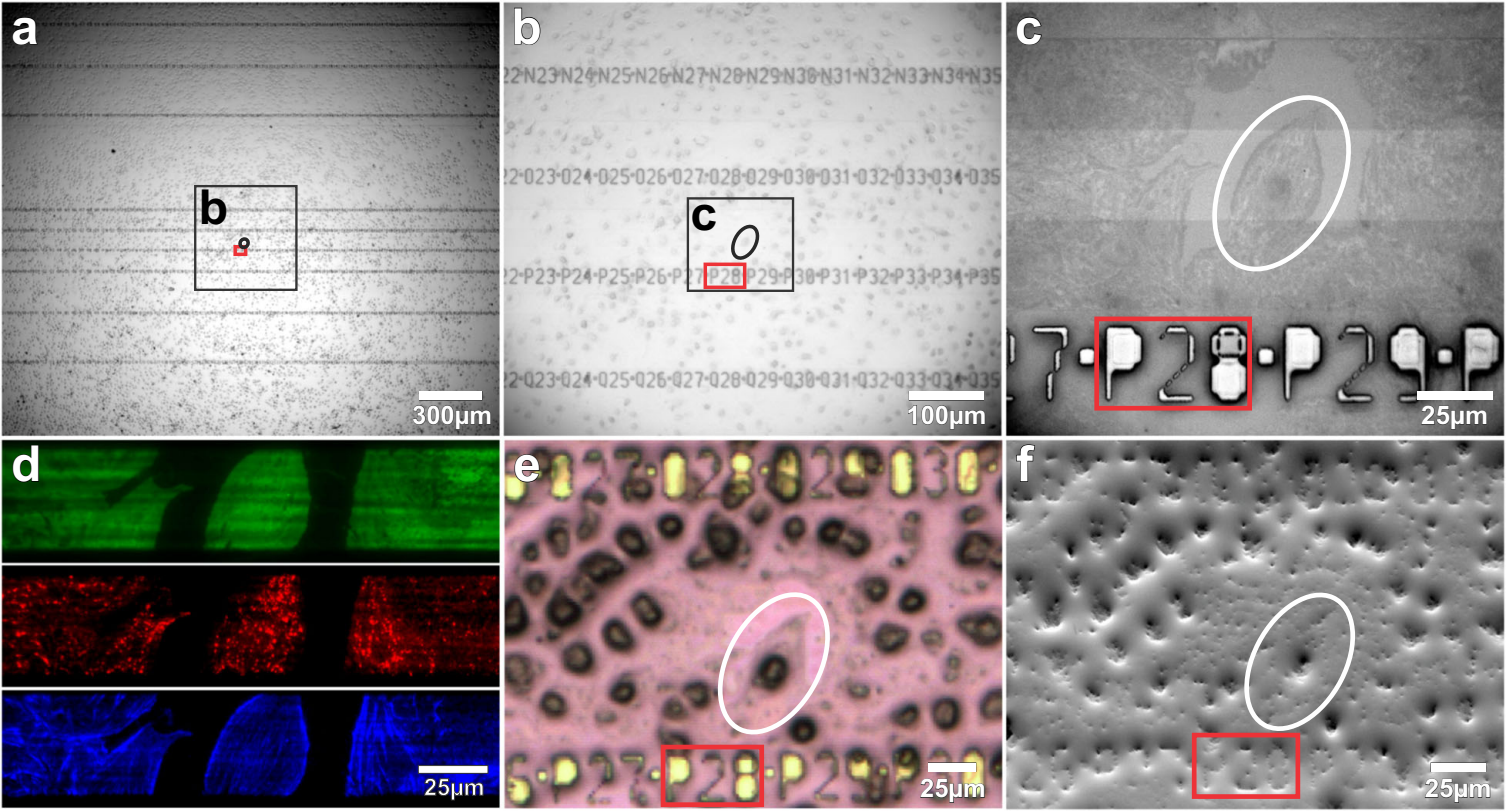
Landmark visibility and localization of region of interest in LM and SEM. **a**–**c** Brightfield image of landmarked chip with LSECs, cell of interest with related landmarks under (**a**) 4×, (**b**) 20× and (**c**) 60× magnification. **d** Chip-based TIRF image of cell membrane (green), protein undergoing endocytosis (red) and actin (blue). **e** Brightfield image after resin coating. **f** SEM image of processed sample inside the FIB-SEM with visible landmarks.

In order to overcome the challenges related to the point of reference location, we designed a special grid-like numbering (landmarks) out of the cladding layer (Fig. 1b, Supplementary Note 1 “Chip fabrication”, Fig. S1) in the form of silicon dioxide pillars (cladding layer). Each point of these landmarks consists of a square with 5 µm side length followed by a letter for a specific waveguide and a continuous numbering system. Each number is 10 pixels in size with a physical pixel size of 2 µm. With a font size of thus 20 µm, high contrast and visibility is provided for light microscopy. While the thickness of the waveguide layer is only 150 nm, the landmark height is 1.5 µm. A layer of resin is needed to stabilize the cell for data acquisition with the FIB-SEM. Resin casting followed by centrifuging the chips in a vertical position at 37 °C can achieve a layer thickness below 1.5 µm, which is sufficient for the landmarks to be visible during SEM imaging (Fig. 1c). There might be minor inconsistencies between individual landmarks (Fig. 2f, Supplementary Note 2 “Landmark visibility”, Fig. S2), but the combination of numbering along the waveguide throughout the full photonics chip and the shape of the cells gives enough guidance to return to the correct cell of interest. This allows precise and fast localization of the cell of interest in the SEM (Fig. 2, Supplementary Note 2 “Landmark visibility”).

**Fig. 3:**
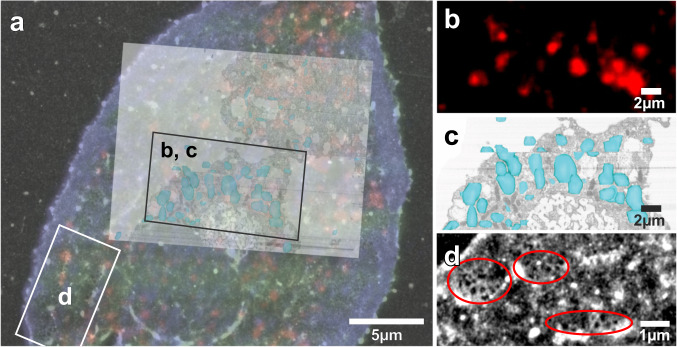
Chip-based TIRF and dSTORM imaging combined with FIB-SEM of LSECs, sample 1. **a** Chip-based TIRF and dSTORM overlay of a LSEC including a correlation to the corresponding FIB-SEM datastack. **b** Chip-based TIRF showing AF488-FSA (in red) internalized in vesicles upon 2 h incubation with the cells. **c** 3D reconstruction of lysosomes based on the FIB-SEM datastack and the *x*/*y* re-sliced imaging plane (manually segmented in light blue). **d** chip-based dSTORM of the cell surface displaying fenestrations (in encircled areas).

**Fig. 4:**
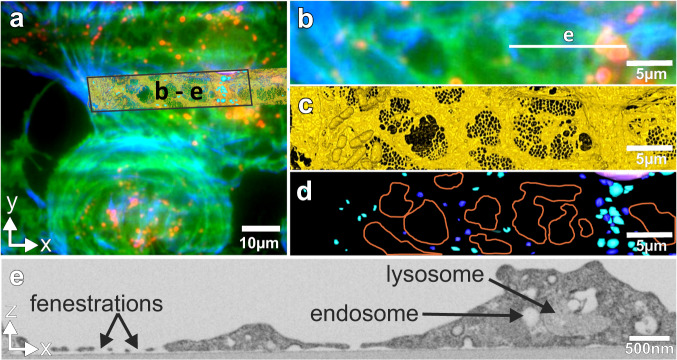
Chip-based TIRF imaging combined with FIB-SEM of LSECs, sample 2. **a**, **b** Three channel chip-based TIRF, labeling actin (Phalloidin, in blue), cell membrane (CellMask, in green) and FSA after 2 h endocytosis (AF647-FSA, in red). **c** 3D reconstruction of FIB-SEM datastack viewed from the *x*/*y* plane showing fenestrations, **d** 3D projected overlay of SEM reconstruction showing endosomes (manually segmented in dark blue) and lysosomes (manually segmented in light blue), as well as graphical representation of the sieve plates (orange line). **e** FIB-SEM cross-section, *x*/*z* plane from Supplementary Video 1, corresponding to the red line in **b**. Correlation between (**b**) and (**d**) indicate that the endocytosed FSA is specifically located in the lysosomes.

To demonstrate the photonic-chip-based CLEM methodology and its applicability in cell biology, we used primary rat liver sinusoidal endothelial cells (LSECs). These cells are a very specialized type of endothelium differing morphologically and functionally from other endothelial cells, and represent a perfect tool for nanoscopy^21^. Their extensive and thin cytoplasm contains many trans-membrane pores (fenestrations) of approximately 50–200 nm in diameter, typically grouped together in “sieve plates”^22,23^. In a normal liver, the fenestrations function as a sieve, retaining the blood cells in the sinusoidal lumen, and allowing molecules smaller than the diameter of the fenestrae, such as metabolites, plasma proteins, pharmaceutical drugs, lipoproteins and small chylomicron remnants, viruses and exosomes to pass through and access the underlying hepatocytes^23,24^. Besides their filtration role, LSECs have an extraordinary endocytic function, effectively clearing the blood from a variety of physiological and non-physiological waste macromolecules and nanoparticles^25^. The cells recognize and internalize circulating macromolecules, which are then rapidly trafficked and efficiently degraded in the endo-lysosomal compartment. Formaldehyde-treated serum albumin (FSA) is a functional marker of LSEC receptor mediated endocytosis and the endo/lysosomal compartment^25–30^. Here, we used the photonic chip to demonstrate its applicability for visualization and correlation of the LSEC fenestrations and endocytosis using TIRF and dSTORM, and subsequently, FIB-SEM. To this end, LSECs were seeded directly onto the fibronectin-coated photonic chips following standard protocols used for cell attachment^31,32^. LSECs preserve their morphology (fenestrations) and functionality (endocytosis) when seeded on various fibronectin/collagen coated substrates, e.g., plastic, glass coverslips, glass-bottom dishes, polycarbonate transwells, PDMS, and photonic chips^11,29,30,33^. The cells were incubated with FSA tagged with AF647. Two hours post incubation, the cells were fixed to arrest their status and prepared for TIRF/dSTORM imaging. The cells were then stained with Phalloidin 488 labeling the actin cytoskeleton, and with CellMask Orange labeling the membrane envelope. These dyes can penetrate the phospholipid layer of the membrane or enter the cell membrane without the need of permeabilizing agents, due to the sufficient but insignificant loss in membrane integrity created by the formaldehyde dissolving some lipids in the membrane bilayer^34^. This was a critical aspect in order to achieve the best ultrastructural preservation possible for FIB-SEM. Moreover, these dyes are also compatible with fixation, thus allowing us to also overcome the challenge with the loss of fluorescence upon the use of chemical fixation. Further, the cells were thinly embedded in a resin^35^ with appropriate viscosity in order for the landmarks on top of the photonic-chips to remain visible through the resin layer (Fig. 2e, f, Supplementary Note 2 “Landmark visibility”). The landmarking enabled us to precisely target individual cells and acquire them with a voxel size of 5 × 5 × 8 nm^3^ (Figs. 3 and 4, Supplementary Movie S1). As a result of imaging using chip-based TIRF and dSTORM, followed by FIB-SEM of selected cells of interest, we were able to visualize and correlate in 3D the structural organization of the fenestrations on LSECs, and to correlate the endocytosed cargo with its specific endo-lysosomal compartments^30^, shown here for two distinct samples in Figs. 3, 4. Figure 2 shows the landmarked waveguide carrying the LSECs, where multi-color chip-based TIRF images were acquired to visualize plasma membrane (in green), endocytic vesicles (in red), and actin filaments (in blue). Brightfield and fluorescence signals were used to scan at low magnification (4× or 20×) for targeting highly endocytic cells with most intact cytoplasm, see Supplementary Note 3 “Large FOV and multiplexing”, Figs. S3 and 2a, b. After locating a cell of interest, brightfield (Fig. 2a), chip-based TIRF (Figs. 2d, 3b, 4a, b) or chip-based dSTORM images (Fig. 3d) were taken at higher resolution simply by changing the detection objective to 60×, 1.2 NA (Figs. 3a–c, S3). Figures 3 and 4 show that the structures identified by light microscopy (Figs. 3a, 4a, b) could be correlated to endosomes/lysosomes and fenestrations in the electron microscopic images (Fig. 4c, d). This was visualized after thresholding in Amira (FEI) and looking at the *x*/*z* surface (for fenestrations) and at the x/y slicing plane (for the internal organelles). The endocytosed FSA visualized by TIRF (red in Fig. 4a, b) was correlated to its internalization compartments, the endosomes and lysosomes (dark blue and light blue in Fig. 4d), identified based on their electron dense appearance under EM. On the other hand, the non-resolved dark-green areas on the cell membrane seen in TIRF (Fig. 4a, b) were identified as the sieve plates containing dozens of fenestrations, as demonstrated through dSTORM (Fig. 3d) and direct correlation after performing FIB-SEM (Fig. 4c).

Imaging on photonics chips opens up a new screening platform for high-resolution light microscopy imaging. Here we have shown that the photonic-chip platform can be combined with electron microscopy in a correlative workflow. From the light microscopy side, the photonic chips offer the possibility to acquire not just diffraction limited TIRF imaging, but also dSTORM on chip, taking again advantage of the huge field of view for screening possibilities. For CLEM, landmark fonts with 2 µm physical pixel size were used. The stepper-free optical lithographic process can be optimized to allow 1 µm pixel size or less, offering accurate localization, while more advanced fabrication methods (such as electron beam writing) can further extend the localization precision. Using microwave-assisted processing and minimal resin embedding allows for a rapid and efficient sample processing, thus fast and easy targeting. 3D-FIB-SEM data is then acquired of the same region imaged by nanoscopy to further detail information about three-dimensional internal structures. Correlative light and electron microscopy suffers from rather low throughput. Landmarked photonic-chip technology has the potential to be used for targeting of multiple cells of interest and acquiring 3D-EM dataset automatically in the future. Moreover, the waveguide chip can be pigtailed using an optical fiber (see Fig. S4) to deliver laser illumination directly inside the electron microscope^36^. This photonic chip-based integrated CLEM approach would be applicable where, e.g., no additional EM processing is required^37,38^. Thus, chip-illumination introduces new possibilities of performing chip-based dSTORM^4,5^, ESI^4^, SOFI and TIRF-SIM^16^ directly inside the EM.

## Methods

### Cell extraction

For the isolation of the primary liver sinusoidal endothelial cells (LSECs), we used Sprague Dawley, Crl:CD(SD), male rats, age 2–3 months, purchased from Charles River, Sulzfeld, Germany. The cells were prepared by collagenase perfusion of the liver, low speed differential centrifugation and Percoll gradient sedimentation^31^, followed by the depletion of Kupffer cells (KC) by seeding the nonparenchymal fraction onto plastic culture dishes. Compared to the LSECs, the KCs adhere strongly to plastic, thereby enriching the LSEC fraction.

### Sample preparation

PDMS (150 µm thick) (Sylgard 184, Dow Corning) was prepared by spin coating in a Petri dish, and square frames of approximately 1.5–2 cm side length were cut and deposited on the waveguide chips. After coating the area within the PDMS frame with fibronectin, the cells were seeded and incubated for 1 h at 37 °C. Non-attached cells were removed by washes with PBS, and the cells incubated for another hour. For endocytosis, cells were incubated with fluorescently-labeled formaldehyde-treated serum albumin (AF647-FSA, 50 µg/ml) for 15 min at 37 °C. Unbound AF647-FSA was washed off with PBS and the cells incubated at 37 °C for 2 h. The cells were pre-fixed with 2.5% glutaraldehyde, 4% formaldehyde and 0.05% malachite green in 0.1 M cacodylate buffer for 15 min at RT. The plasma membrane was stained by incubating the cells for 10 min at RT with CellMask Orange 561 (1.25 ng/ml in PBS). The actin filaments were stained by incubating cells for 45 min at 37 °C with Alexa Fluor 488 Phalloidin (1:40 dilution in PBS). For the imaging buffer, 22.5 µl of a water-based oxygen scavenger system solution (based on glucose oxidase and catalase, Sigma-Aldrich) was mixed with 30 µl PBS^39^. For dSTORM measurements, 2 mM cyclooctatetraene and 95 mM mercaptoethylamine (Sigma-Aldrich) were added to the imaging media^11^. After adding the imaging media to the cell chamber, the chamber was sealed by gently pressing down a coverslip against the PDMS frame.

### Waveguide imaging

The waveguides with the fixed cells were imaged on a custom-made system. Three lasers with wavelengths at 488 nm (Oxxius), 561 nm (Cobolt) and 660 nm (Cobolt) had their beam width increased with two lens telescope systems to fill the back aperture of the in-coupling objective (Olympus 50×, 0.5 NA). Optical elements as neutral density filters to reduce the minimum laser power were used when necessary. The in-coupling objective and the sample holder were mounted on two separate multi axis stages (Thorlabs), the coupling objective stage disposing of piezo motors and the sample stage of a vacuum mount (more details and schematic diagram under Supplementary Note 4 “Waveguide setup”, Fig. S4). After placing the sample at the vacuum mount, laser light was focused onto the waveguide edge with the coupling efficiency optimized through the piezo motors. Brightfield and fluorescence images from the waveguide surface were collected by 4×, 20× and 60× (1.2 NA, water immersion) objectives (Olympus) at a modular microscope system (Olympus BXFM). The microscope was equipped with a filter wheel (Thorlabs, through a home-made adaptor) with a notch and a long pass filter (AHF) for each laser wavelength, where the magnification-free tube directed the light to a sCMOS camera (Orca-Flash 4.0, Hamamatsu). As the utilized waveguide geometries are multimode at the excitation wavelengths, the illumination pattern by the coupling objective at focal distance is highly inhomogeneous. This interference between the modes can be heavily reduced towards a more homogeneous distribution by oscillating the coupling objective along the input facet of the waveguide (Fig. S5). The chip-based images presented in this work were performed with rather narrow waveguides of 25 and 70 µm width, where spurious interference patterns are visible. This can be attributed to the limited travel range of the piezo stage holding the coupling objective and can be optimized, as seen in other publications^11,12,15^. More information is provided in the Supplementary Note 5 “Homogeneous illumination with multimode waveguides”. It should further be mentioned that the mixing of waveguide modes will not influence the evanescent field’s penetration depth, the latter being constant (less than 1% variation) for different modes and waveguide widths at fixed illumination conditions. The change in polarization or wavelength will however affect the evanescent field decay. Supplementary Note 6 “Evanescent field penetration depth” and Fig. S6 provide values and more details to such calculations. After imaging, the coverslip and PDMS frame were removed and the chips returned to fixative until further processing (see Supplementary Note 7 “Work flowchart”, Fig. S7). To characterize the optical resolution of the setup, fluorescent beads were imaged indicating a resolution of 390 ± 15 nm for TIRF at 60×/1.2 NA magnification, at an emission wavelength of ca. 680 nm (theoretical value: 346 nm). The dSTORM measurement of a monomolecular dye layer with the same objective and magnification points towards a potential resolution of 22 nm. More information and plots to the resolution measurements are available under Supplementary Note 8 “Waveguide imaging resolution” and Fig. S8.

### Waveguide processing for electron microscopy

All processing was done in a Ted Pella Biowave with a temperature control unit (Ted Pella Inc.)^5,40^. Because the photonic-chips were overheating upon microwave processing, the samples were placed directly on the control unit, set to 4 °C, and the vacuum chamber was inverted on top. To stabilize the cells further after light microscopy the samples were processed for 14 min (2 min vacuum on-off-on-off-on-off-on, 100 W) in fixative and washed twice with 0.1 M cacodylate buffer. Post-fixation was done with 1% Osmium tetroxide, 1% K_3_Fe(CN)_6_ in 0.1 M cacodylate. The cells were post-stained with 1% tannic acid and 1% uranyl acetate. Samples were then dehydrated in increasing ethanol series (30%, 50%, 75%, 90% and 2 × 100%) and embedded in increasing amounts of Durcupan (30%, 50%, 75%, 90%). To be able to remove as much resin as possible, the resin exchange steps were increased to 90% Durcupan in EtOH and not 100% Durcupan. The chips were centrifuged for 30 min at 37 °C in a vertical position to further remove excess resin, and polymerized in the oven for 96 h at 60 °C. The chips were then cut to a final size of 1 cm^2^ to fit the SEM stubs.

### FIB-SEM on cell-monolayer

The photonic chip with minimally embedded cells and landmarks on the surface was mounted on a SEM stub (6 mm length, Agar Scientific) using a conductive carbon sticker (12 mm, Plano GmbH, Germany). To reduce the amount of charging, the samples were surrounded by silver paint and gold coated for 180 s at 30 mA in a sputter coater (Quorum, Q150RS). The samples were introduced into the Crossbeam 540 (Carl Zeiss Microscopy, Germany). Light microscopy images of the landmarks were used to target the correct cell inside the FIB-SEM. The FIB was used at 15 nA to mill a trench and expose a cross-section through the cell. A current of 3 nA was used for polishing the cross-section before imaging. For imaging, the FIB milling was operated with 1.5 nA, the SEM imaging and the FIB milling operating simultaneously^41^. The SEM images were acquired at 1.5 kV with the Energy selective Backscattered (EsB) detector with a grid voltage of 1100 V, analytical mode at a 700 pA current, setting the dwell time and line average to add up to 1.5 min per image. The final dataset was acquired with 5 × 5 nm^2^ pixel size and a slice thickness of 8 nm.

### Image post-processing

The following image post-processing steps were performed in Fiji^42^. The image stacks were aligned using TrackEM2^43^, cropped and inverted. Following a smoothing step, the 3D segmentation of the fenestration was performed using thresholding in Amira (Thermo Fisher). The individual organelles were segmented in IMOD^44^.

### Statistics and reproducibility

Figures 2–4 are representative of 3–5 separate experiments with cells isolated from different rats. Approximately 30–40 cells from different locations on multiple waveguides were imaged with light microscopy during each experiment. 4 samples have been analyzed with FIB-SEM, of which two were thoroughly analyzed in terms of 3D visualization and correlation.

### Ethical compliance

Sprague Dawley, Crl:CD(SD), male rats (Charles River, Sulzfeld, Germany), 2–3 months of age (body weight 150–300 g) were used for this study. Prior to liver cell isolation, the rats were anesthetized with a mixture (ZRF-mix) of zolazepam/tiletamine hydrochloride 12.9/12.9 mg/mL (Zoletil forte vet, Virbac, Norway), xylazine 1.8 mg/mL (Rompun, Bayer Nordic, Norway) and fentanyl 10.3 μg/mL (Actavis, Norway). All methods were carried out in accordance with relevant guidelines and regulation protocols, and approved by the Norwegian Food Safety Authority (approval ID: 8455). All experimental protocols and animal handling were approved by and carried out according to local authorities (Department of Comparative Medicine).

## Supplementary information

Supplementary Information

Description of Additional Supplementary Files

Supplementary Video 1

## Data Availability

Data that support the findings of this study are stored in servers and are available on reasonable request, see author contributions for specific data sets.
