# Supplementary material for: Photonic-chip assisted correlative light and electron microscopy
Source: Commun Biol. 2020 Dec 7;3:739. doi: 10.1038/s42003-020-01473-4 (PMC7721707; doi:10.1038/s42003-020-01473-4)
Supplement: Supplementary file 1 — Supplementary Information [file 42003_2020_1473_MOESM1_ESM.pdf]

Photonic-chip assisted correlative light and electron microscopy

Supplementary Information

Jean-Claude Tinguely, Anna Maria Steyer, Cristina Ionica Øie, Øystein Ivar Helle, Firehun Tsige Dullo, Randi Olsen, Peter McCourt, Yannick Schwab, Balpreet Singh Ahluwalia

Supplementary Note 1 "Chip fabrication"

The photonic chips were manufactured through standard photolithography at the Institute of Microelectronics Barcelona (IMB-CNM, Spain). The scheme in Figure S1 depicts only a small section of a single waveguide for better visualization. 1 mm thick, 4 inch silicon wafers were used as substrates (Figure S1A). First, a SiO_2 layer with a thickness of approximately 2 µm was thermally grown on the surface (Figure S1B). 150 nm of Si_3N_4 were deposited using low-pressure chemical vapour deposition (LPCVD) at 800°C (Figure S1C) followed by thermal annealing at 600°C. Photolithography was employed to pattern the waveguide geometry, with reactive ion etching (RIE) defining the etch depth (Figure S1D). The remaining photoresist was removed, and 200 nm SiO_2 followed by 100 nm polycrystalline silicon deposited by plasma-enhanced chemical vapour deposition (PECVD) at 300°C. These 300 nm of material were patterned by RIE (the first 200 nm) and hydrofluoric acid (the last 100 nm) as channels beside the waveguides (10 µm gap between waveguide and absorbing layer walls) (Figure S1E). Such structures are meant to reduce cross-illumination between neighboured waveguides, of special importance for shallow rib waveguides with low light confinement. For strip etched, multimode waveguides, they mostly help towards raising the total height of the landmark structures. Finally, 1.5 µm of SiO_2 were deposited for the cladding layer by PECVD. Taller layers were avoided to prevent high stress in the layer and because of the slow deposition process. Photolithography is performed with RIE down to 100 nm of material and hydrofluoric acid for the remaining SiO_2 thickness. An area of 13 x 14 mm was left exposed at the center of the chip with landmark structures placed on the absorbing layer along the direction of light propagation (Figure S1F). The wafers were diced with a precision saw to form chips of approximately 2-3 cm side length and the edges with waveguide inputs polished with abrasive discs of 1 µm and 0.5 µm grit size (Buehler). Optical characterization of 1.5 µm wide waveguides exhibited less than 1dB/cm loss at 660 nm, approximately 2 dB/cm loss at 561 nm and approximately 10 dB/cm loss at 488 nm[1].

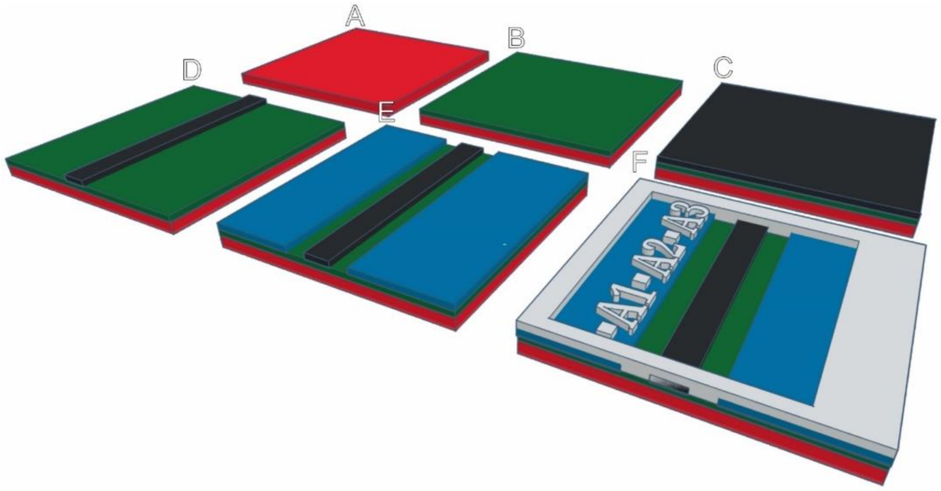

Figure S1: Scheme of fabrication steps of the waveguide chips. A) 1 mm thick silicon wafer. B) Approximately 2 µm thick SiO_2 created through thermal oxidation. C) Deposition of 150 nm thick Si_3N_4. D) Photolithographic patterning followed by etching of Si_3N_4. Waveguide widths for imaging range between 25 to 500 µm. E) Deposition and patterning of absorbing layer consisting of 200 nm thick SiO_2 (bottom) and 100 nm thick polycrystalline silicon (on top). F) Deposition and patterning of 1.5 µm thick SiO_2 cladding for imaging regions and landmarks.

Supplementary Note 2 “Landmark visibility”

Landmarks were used as a coordinate system in optical (Figure S2 A-C) and electron microscopy (Figure S2 D-E). Figure S2 A-C shows the high contrast of landmarks under brightfield illumination at the full field of view of 4x, 20x, and 60x magnification, respectively. High contrast and thus visibility allow for easy position readout. Figure S2 D-E are scanning electron microscopy images of different chips after the resin step, showing resin thickness variations that can occur after centrifugation and polymerization. A prerequisite for optimal resin thickness, thus landmarks visibility, was to centrifuge the chips in vertical position at 37°C for 30 min. Despite different degrees of visibility, the landmarks can still be identified in the scanning electron microscope.

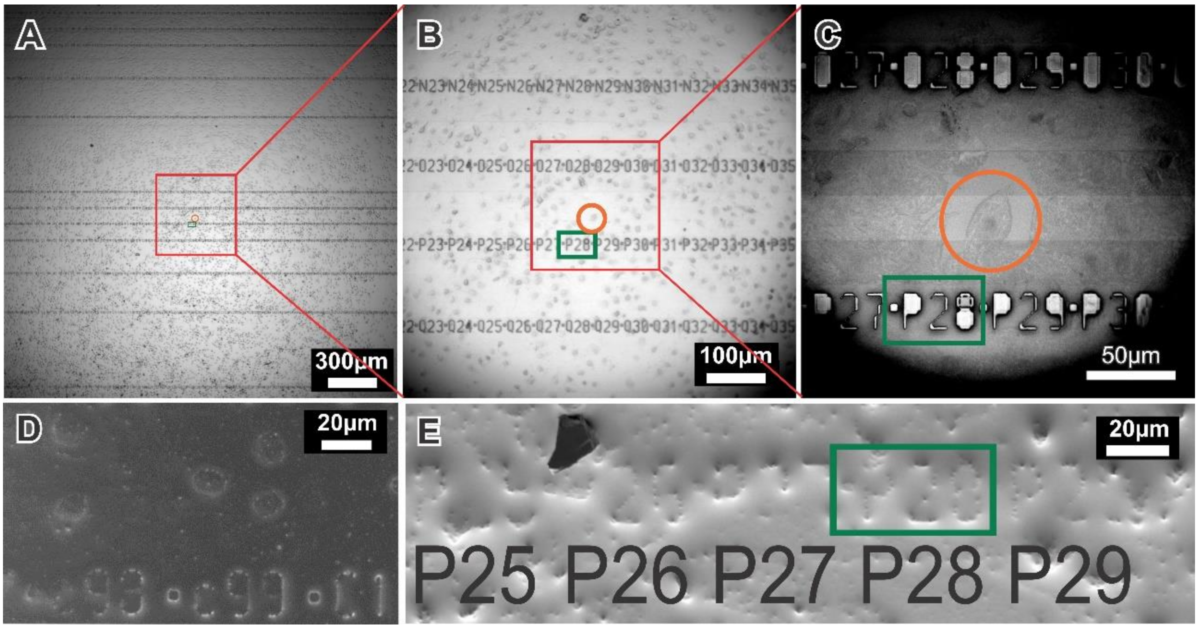

Figure S2 A-C: Landmark visibility under optical brightfield microscope. A) 4x, B) 20x and C) 60x magnification, full field of view. D-E) Resin embedded chips (2 different samples) under the scanning electron microscope.

Supplementary Note 3 “Large FOV and multiplexing”

Making use of a waveguide chip as a substrate and illumination source decouples the typical dependency between excitation and collection optics. It offers the generation of evanescent field without the necessity of a specialized high magnification/numerical aperture TIRF lens, enabling free choice of imaging objective. This property is of advantage for large field of view TIRF-imaging applications. Here, an isolated LSEC was identified and selected by only using the signal from the membrane stain emission channel under 4x magnification, after which a 60x multi channel image was taken (Fig. S3).
For conventional, objective-based TIRF setups, multicolor imaging is cumbersome as it requires repositioning of the beam to fulfill the total internal reflection condition when changing between excitation wavelengths. The waveguide setup allows for easy multiplexing, as an achromatic coupling lens will provide the same beam spot for coupling between different laser lines without the necessity of mechanical adjustments. As propagation losses in the waveguide are higher for shorter wavelengths, compensation in the illumination intensity might be necessary. It was however observed that the intensity of the fluorophore stain had at least a similar influence in the signal to noise ratio.

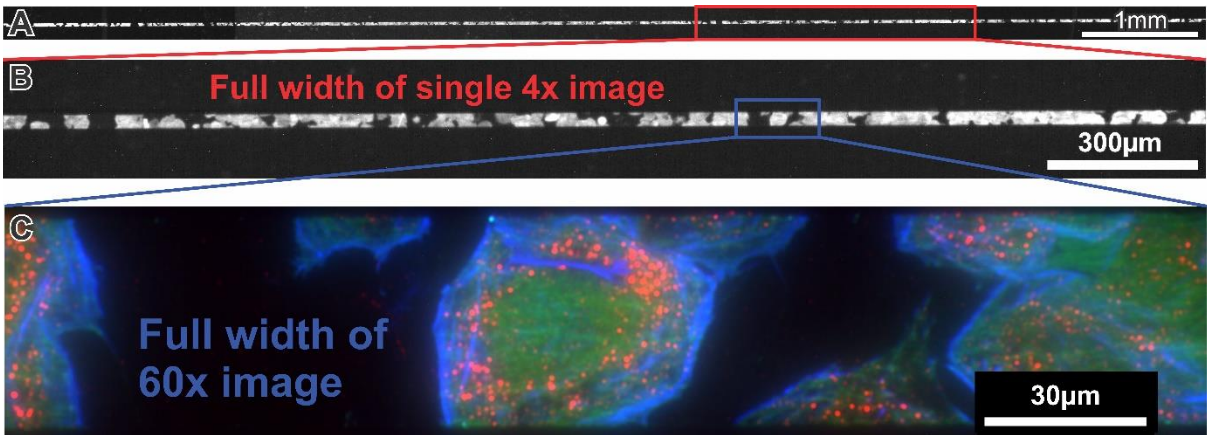

Figure S3: Decoupling of excitation and collection in waveguide-based TIRF imaging allows for simple switch between low magnification overview and high-resolution images. A) ca. 1 cm stitched from 4x 0.1 NA images, showing cell membrane staining. B) Full width of single low magnification image. C) Full width of high-resolution image (60x, 1.2 N.A.), three channel waveguide-based TIRF.

Supplementary Note 4 “Waveguide setup”

A scheme of the setup is shown in Figure S4 A) Laser beams at three conventional bioimaging wavelengths (561 nm 500 mW and 660 nm 500 mW- Cobolt, 488 nm 200 mW - Oxius) are expanded to slightly overfill the back aperture of the coupling objective (Olympus, 50x / 0.5 NA). The laser beams are aligned using dichroic mirrors (Edmund Optics) towards the coupling objective mounted on a 3 axis flexure stage featuring piezo motors (Thorlabs Nanomax). The waveguide chip is mounted on a separate 3 axis flexure stage (Thorlabs Nanomax) with a vacuum chuck (Thorlabs) to hold the chip. A modular microscope setup (Olympus BXFM) mounted on motorized translation stages for two axis translation (Thorlabs) provides brightfield illumination and acquires images at different magnifications (4x, 20x / 0.45 NA, 60x / 1.2 NA water immersion, all Olympus). High magnification is used with a z-piezo system (PIFOC, PI) for fine adjustment. A homemade adapter holds a cage filter wheel (Thorlabs) between the microscope body and the tube lens (1x). For each excitation wavelength, a long pass and a band pass filter were used (488 LP and 520 ± 36 nm, 561 LP and 591 ± 43 nm, 664 LP and 692 ± 40 nm, AHF Analysentechnik), and the images recorded with a sCMOS camera (Hamamatsu Orca Flash).

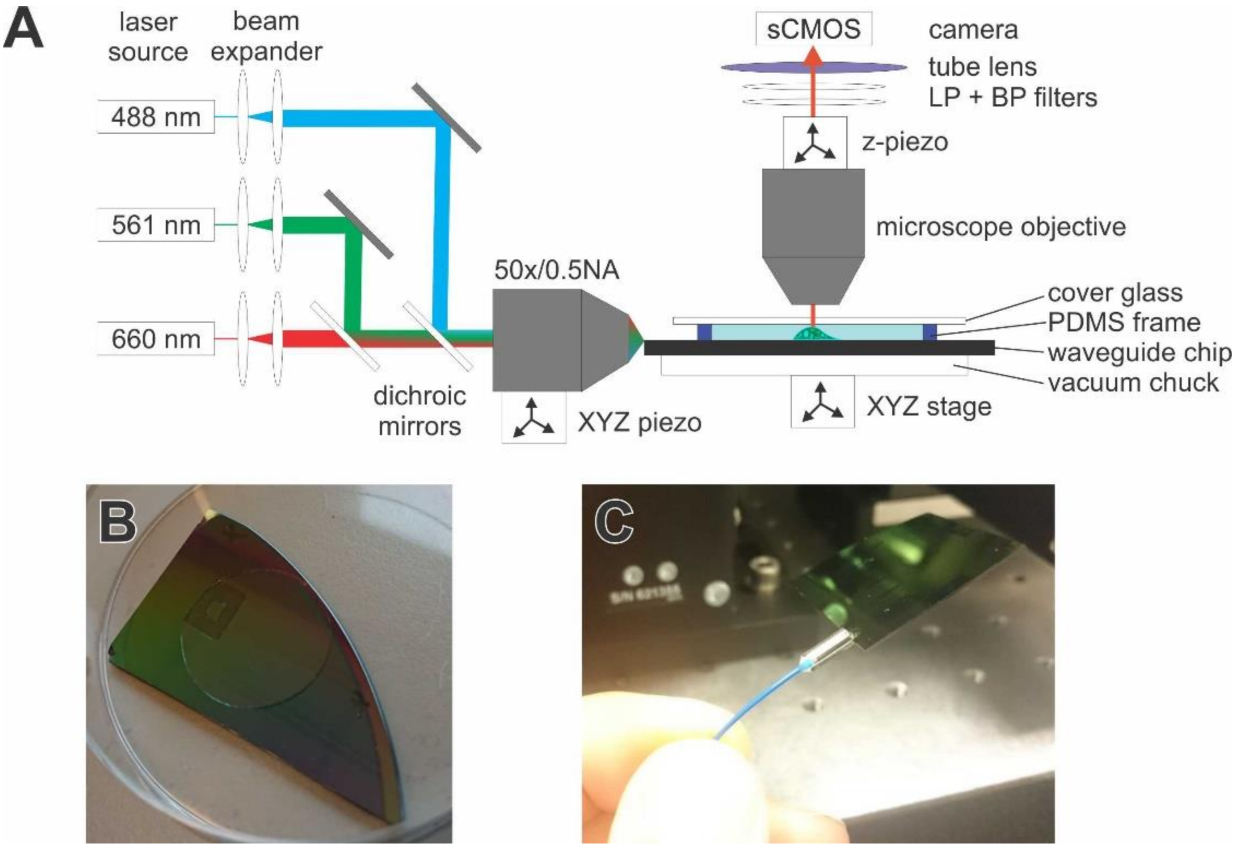

Figure S4: Waveguide setup A) Experimental-set-up for waveguide-based optical micro-/nanoscopy. B) Waveguide chip with mounted PDMS frame and coverslip sealing the imaging region. C) Single-mode fiber pigtailed to waveguide facet as an alternative objective-based coupling method, which could be useful for integrated CLEM.

Supplementary Note 5 “Homogeneous illumination with multimoded waveguides”

Recently, for Si_3N_4 waveguide platform operating at visible wavelengths, a single condition carrying only the fundamental mode was demonstrated using a rib waveguide geometry[3] for 150 nm thick and 1.5 µm wide Si_3N_4 waveguides. Adiabatic tapering can increase the width of the structures for imaging, but required taper length for widths of hundreds of micrometers would be of several millimetres[1]. In this work, we employed a stripe geometry fully etching the waveguide surrounding. For the width of the waveguide used in this work (25 µm), the waveguide supports several modes[2]. The presence of multiple modes creates interference patterns and so uneven illumination on the surface. This can be reduced by scanning the coupling beam over the waveguide width (Figure S5) and averaging the mode beating pattern generating a uniform image[4]. This allows for the use of waveguide structures of several hundreds of micrometers in width. In Figures 2d and 4a, striations from the mode beating pattern are still visible. Those can be attributed to the relatively narrow width of the waveguide (25 µm), but also to the limited travel range of the piezo motor (20 µm) utilized to scan the input coupling. Wider waveguides, meaning more modes, and a stage with longer travel range will ideally provide striation free images, as it can be seen in previous publications[1, 3, 4]. Alternatively, a galvo mirror can deflect the beam on the back aperture of the coupling objective, scanning the waveguide input at high frequency. This will require only a single or a small number of images compared to the translation stage used in this work, where typically 100 images were acquired for averaging. However, as it is difficult to tune the galvo mirror for an ideal deflection angle, it comes at cost of lower laser intensities reaching the waveguide.

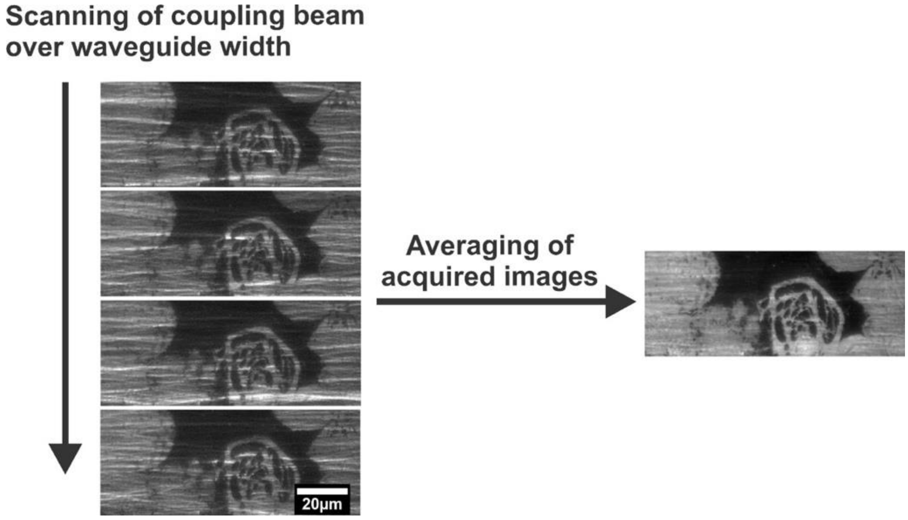

Figure S5: Left: Multimode waveguides provide uneven illumination through interference patterns between modes. These patterns can be varied when moving the coupling beam along the input facet. Right: Averaging images of these mode variations results provide an almost homogeneous illumination pattern.

Suppplementary Note 6 "Evanescent field penetration depth"

Simulations were performed to visualize the decay of the evanescent field above the waveguide. The geometry consisted of a 150 nm thick silicon nitride structure with different widths lying on a silicon dioxide substrate and surrounded by water. The software Fimmwave was used to calculate the effective refractive index of the fundamental TE and TM modes, as well the highest TE mode found for a given width (Figure S6 A). With the real part of the effective refractive index, an analytical solution[5] was used to determine the evanescent field decay (Figures S6 A and B). It can be seen in the table of Figure S6 A that the penetration depth, expressed through the decay to 1/e (ca. 37%) of the initial evanescent field intensity, is higher for longer wavelengths and TM polarization. The intensity difference between the polarization states is also higher at longer wavelengths (ca. 30%), and could be explored to modulate the penetration depth through rotation of the polarization of the incoupling beam. When it comes to the span of modes present for, e.g., pure TE polarization, it can be seen that the mode averaging for homogeneous illumination practically does not affect the penetration depth with differences below 1 nm. This negligible difference between the modes is also kept for different waveguide widths.

A

Wavelength (nm)	Polarization	Mode order	Waveguide width (µm)	n_{eff} (Re)	Penetration depth (1/e) (nm)
660	TE	0	25	0.0972	97.21
660	TE	12	25	0.0977	97.72
660	**TM**	0	25	0.1243	124.31
660	TE	0	100	0.0972	97.20
660	TE	40	100	0.0975	97.50
660	**TM**	0	100	0.1243	124.29
488	TE	0	100	0.0624	62.40
488	TE	40	100	0.0625	62.48
488	**TM**	0	100	0.0739	73.88
660	TE	0	400	0.0972	97.20
660	TE	106	400	0.0973	97.33
660	**TM**	0	400	0.1243	124.29

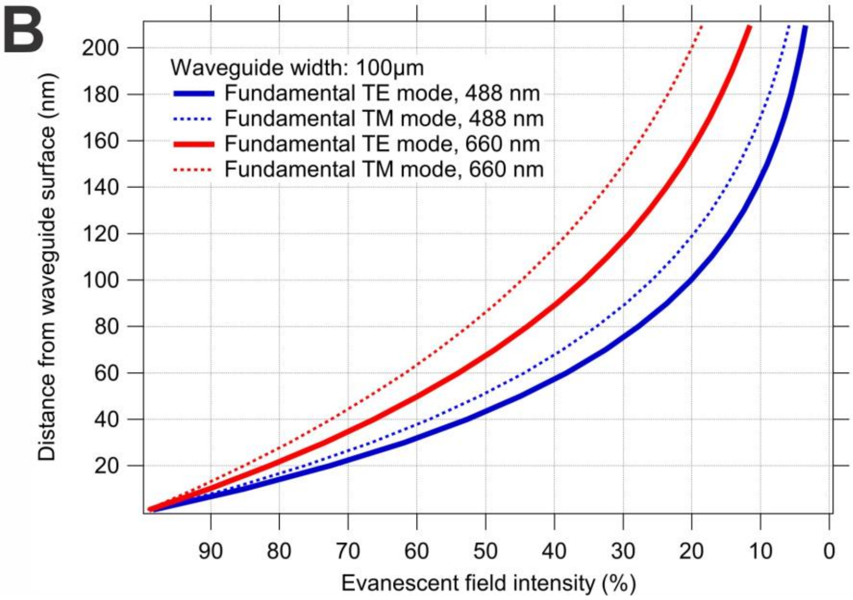

Figure S6: Simulations for the evanescent field decay in a 150 nm tall silicon nitride waveguide. A: penetration depth for different widths, polarization and wavelengths. B: Evanescent field decay for fundamental modes at two different wavelengths and polarization states.

Supplementary Note 7 “Work flowchart”

The flowchart in Figure S7 provides an overview of the processing steps from chip preparation, cell seeding and staining, light microscopy imaging, sample preparation for electron microscopy and FIB-SEM imaging.

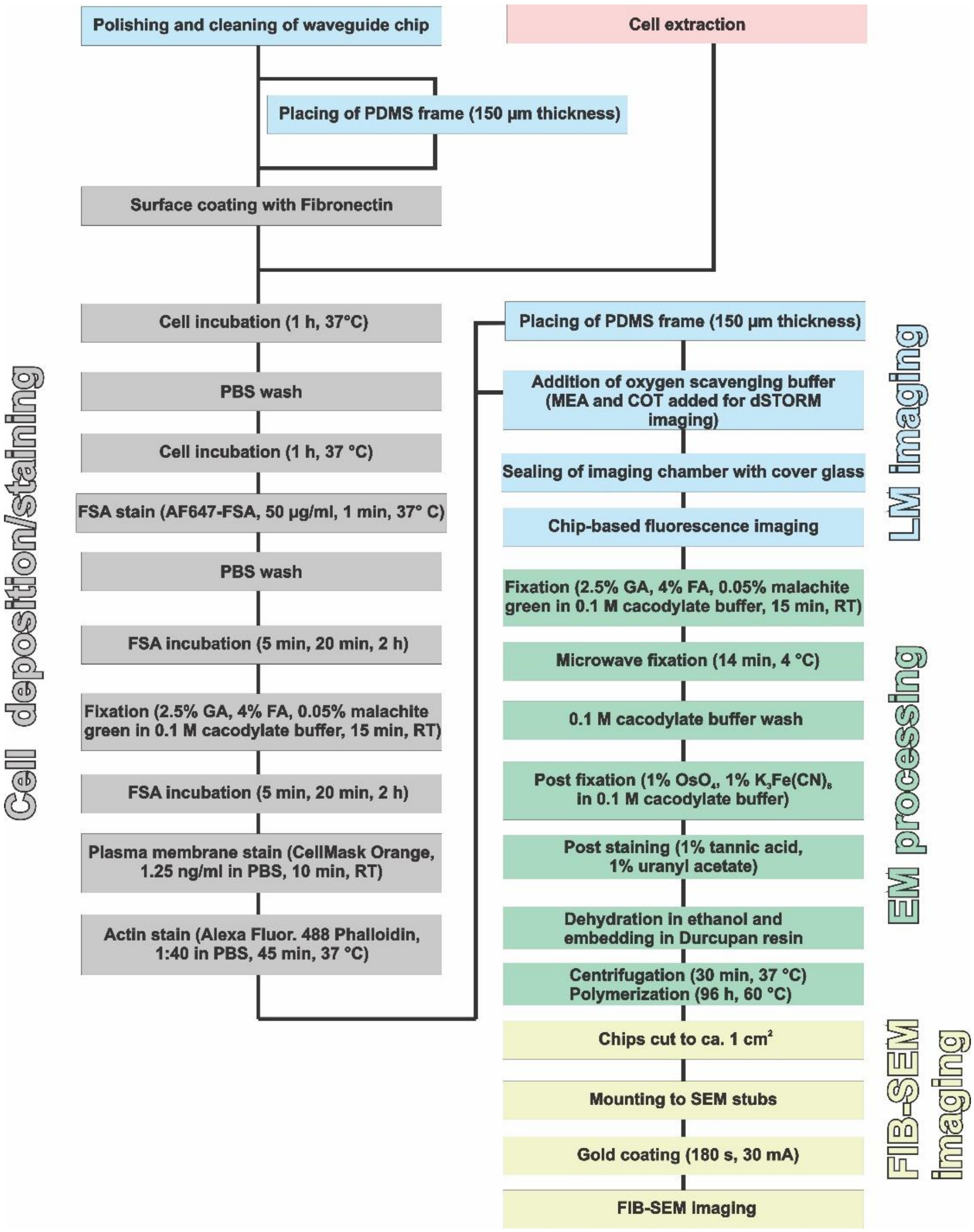

Figure S7: Work flowchart.

Supplementary Note 8 “Waveguide imaging resolution”

To benchmark the resolution for the chip-based TIRF images, fluorescent beads with a diameter of 200 nm (TetraSpeck microspheres, Invitrogen) were deposited on a chip surface, excited at 660 nm and imaged with the 60x/1.2 NA water immersion objective used for the cell experiments. Figure S8 A shows a section of an acquired fluorescence image, with S8 B demonstrating how three of the indicated line profiles were fitted with a Gaussian curve to estimate the resolution through the fit’s full width at half maximum (FWHM). The six points marked in S8A provide an averaged resolution of 390 nm with a standard deviation of 15 nm, the theoretical diffraction-limited resolution for the used objective at the emission wavelength of ca. 680 nm being 346 nm.
For the achievable resolution in the dSTORM experiments, a monomolecular layer of fluorophore was used to determine the localization precision of the blinking sites. For this end, 100 µl of a PBS solution containing 100 nm streptavidin conjugated Alexa Fluor 647 (Invitrogen) was mixed with 100 µm 0.1% poly-L-lysine solution. After incubating at the chip surface for 20 minutes, three wash steps with distilled water remove the excess dye on the chip. Buffer media is added, and the sample is ready for measurements after placing a coverslip on top. Figure S8C presents the frequency of the different dimensions localized with the algorithm of the ThunderSTORM plugin for Image J[6], indicating a potential localization of 9.4 nm or a resolution of 22.1 nm at FWHM for the 60x/1.2 NA objective.

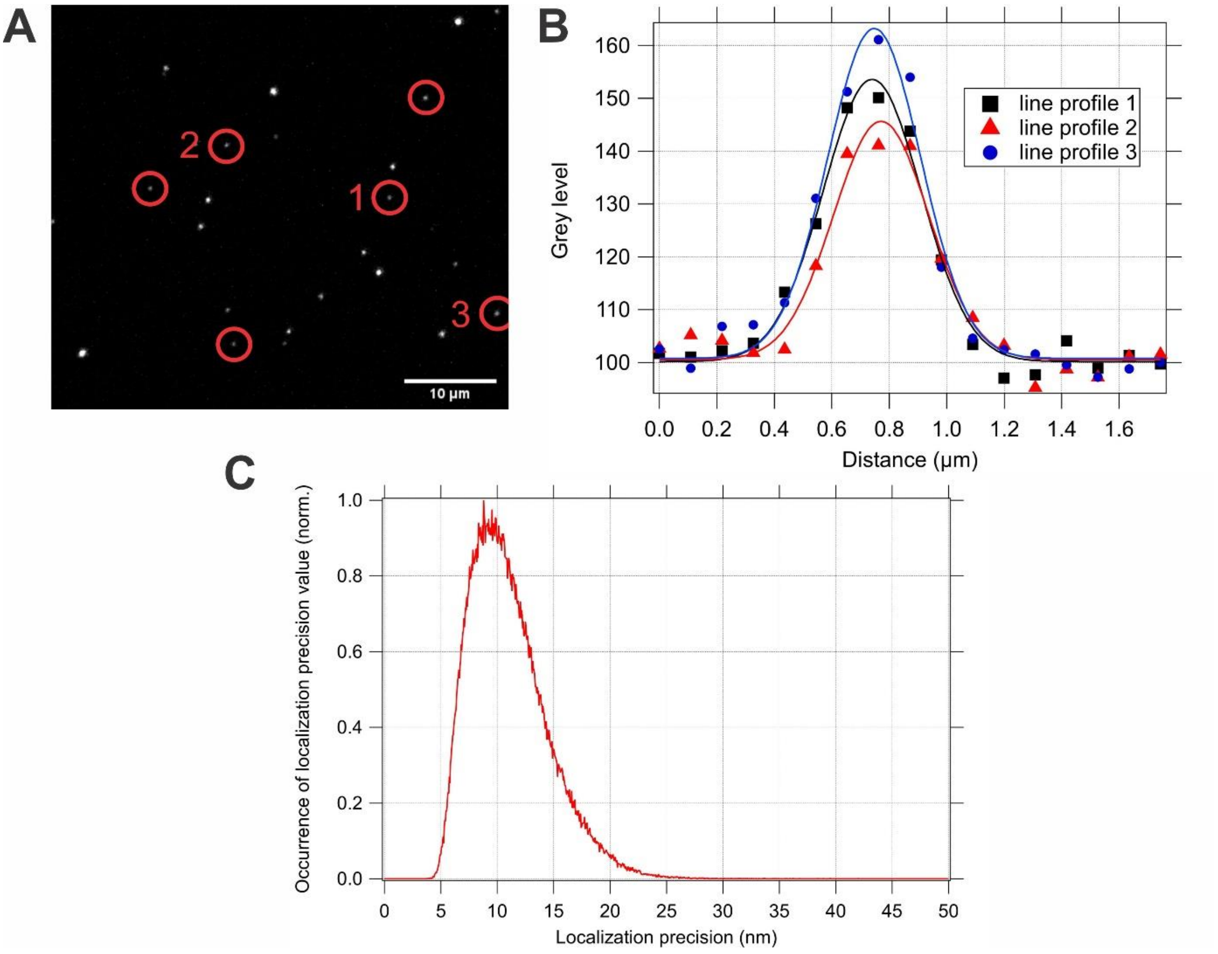

Figure S8: A: Section of chip-based TIRF image of 200 nm fluorescent beads. B: Line profile and Gaussian fit of three beads marked in A, where the resolution is obtained through the FWHM. C: Localization precision of a monomolecular dye layer to obtain the achievable resolution with dSTORM experiments.

Supplementary Information References

1. Tinguely, J.-C., Helle, Ø.I. & Ahluwalia, B.S. Silicon nitride waveguide platform for fluorescence microscopy of living cells. *Opt Express* **25**, 27678-27690 (2017).
2. Dullo, F.T., Tinguely, J.C., Solbo, S.A. & Helleso, O.G. Single-Mode Limit and Bending Losses for Shallow Rib Si3N4 Waveguides. *Ieee Photonics J* **7** (2015).
3. Diekmann, R. et al. Chip-based wide field-of-view nanoscopy. *Nature Photonics* **11**, 322-+ (2017).
4. Helle, Ø.I., Coucheron, D.A., Tinguely, J.-C., Øie, C.I. & Ahluwalia, B.S. Nanoscopy on-a-chip: super-resolution imaging on the millimeter scale. *Opt Express* **27**, 6700-6710 (2019).
5. Bertolotti, M., Sibilia, C. & Guzmán, A.M. in Sprinter Series in Optical Sciences, Vol. 206. (ed. Springer) 45-47 (2017).
6. Ovesny, M., Krizek, P., Borkovec, J., Svindrych, Z.K. & Hagen, G.M. ThunderSTORM: a comprehensive ImageJ plug-in for PALM and STORM data analysis and super-resolution imaging. *Bioinformatics* **30**, 2389-2390 (2014).
