# Supplementary material for: Photonic-chip assisted correlative light and electron microscopy
Source: Commun Biol. 2020 Dec 7;3:739. doi: 10.1038/s42003-020-01473-4 (PMC7721707; doi:10.1038/s42003-020-01473-4)
Supplement: Supplementary file 2 — Description of Additional Supplementary Files [file 42003_2020_1473_MOESM2_ESM.pdf]

Description of Additional Supplementary Files

Title: Supplementary Video 1.

Description: High-Resolution FIB-SEM 3D-reconstruction of fenestrations and endolysosomal compartment in one LSEC (Related to Figure 3)
